# Systematic Review of Pharyngeal and Esophageal Manometry in Healthy or Dysphagic Older Persons (>60 years)

**DOI:** 10.3390/geriatrics3040067

**Published:** 2018-10-05

**Authors:** Charles Cock, Taher Omari

**Affiliations:** 1 Department of Gastroenterology and Hepatology, College of Medicine and Public Health, Flinders University, Adelaide 5042, Australia; taher.omari@flinders.edu.au; 2 Department of Human Physiology, College of Medicine and Public Health, Flinders University, Adelaide 5042, Australia

**Keywords:** manometry, aging, esophagus, pharynx, dysphagia

## Abstract

We undertook a systematic review of swallowing biomechanics, as assessed using pharyngeal and esophageal manometry in healthy or dysphagic older individuals aged over 60 years of age, comparing findings to studies of younger participants. PRISMA-P methodology was used to identify, select, and evaluate eligible studies. Across studies, older participants had lower upper esophageal sphincter (UES) resting pressures and evidence of decreased UES relaxation when compared to younger groups. Intrabolus pressures (IBP) above the UES were increased, demonstrating flow resistance at the UES. Pharyngeal contractility was increased and prolonged in some studies, which may be considered as an attempt to compensate for UES flow resistance. Esophageal studies show evidence of reduced contractile amplitudes in the distal esophagus, and an increased frequency of failed peristaltic events, in concert with reduced lower esophageal sphincter relaxation, in the oldest subjects. Major motility disorders occurred in similar proportions in older and young patients in most clinical studies, but some studies show increases in achalasia or spastic motility in older dysphagia and noncardiac chest pain patients. Overall, study qualities were moderate with a low likelihood of bias. There were few clinical studies specifically focused on swallowing outcomes in older patient groups and more such studies are needed.

## 1. Introduction

Dysphagia is increasingly recognized as an important consideration when assessing older patients or community-dwelling older people [1,2,3,4,5]. The consequences of impaired swallowing can impact on both life expectancy and quality of life. Malnutrition, dehydration, pulmonary aspiration, and increased choking risk may result from dysphagia in older persons [1,2,3,4,5], however depression due to impaired quality of life or the social isolation caused by an inability to eat a meal normally, are less well recognized potential consequences [6]. 

Failure to recognize or adequately address swallowing and feeding problems in older individuals could trigger a downward spiral of sarcopenia and frailty leading to impairment of physical function, leading to further swallowing impairment and worsening sarcopenia/frailty. In some cases, sarcopenia may result in or contribute to dysphagia [7,8,9]. Even healthy, community- dwelling, older individuals are “at risk” due to reduced swallowing functional reserve [10], and this applies more so to hospitalized or institutionalized individuals [11]. 

Pharyngeal and upper esophageal sphincter manometry has to overcome a number of technical challenges that relate to the rapidly changing and widely varying pressures across the pharyngoesophageal segment that are accompanied by significant structural asymmetries [12,13,14]. Historically, it is widely regarded that traditional manometry equipment, using water perfusion, even with sleeve sensors, was unable to overcome these challenges [12]. As a consequence, solid-state transducers were developed that produce interpretable pharyngeal and UES results [12]. The most recent iteration of this development employs sensor spacing of 1cm or less and is referred to as high-resolution pharyngeal manometry, or HRPM. However, outcome measurements assessed using this highly advanced technology, as well as its lower resolution predecessors, vary widely. 

Esophageal manometry is used in conjunction with radiology and endoscopy to definitively diagnose major abnormalities of esophageal peristalsis, such as achalasia [15,16,17,18,19]. Technologies have evolved from widely spaced water-perfused or solid-state pressure sensors used with a “pull through” technique to “high-resolution” manometry (HRM) (pressure sensors spaced at 1cm or less). The clinical use of HRM and “esophageal pressure topography”—a “contour map” of esophageal pressures—have markedly enhanced consistency, ease, and accuracy of major disorders of esophageal peristalsis, and are now the standard of care in esophageal motility disorders [17,18,19,20,21,22]. 

The older population, with a higher prevalence of oropharyngeal dysphagia [11] and potentially major disorders of peristalsis [23,24,25], is likely to benefit from any improved clinical utility of manometry technologies. 

The primary goals of the study were to determine differences in manometry in older subjects (healthy volunteers or dysphagia), as compared to that in younger subjects, studied under similar conditions.

## 2. Methods 

The study design was based on the 2015 version of the preferred reporting items for systematic review and meta-analysis protocols (PRISMA-P) [26,27]. The focus of our study was on studies evaluating participants over 60 years of age (either healthy volunteer groups or dysphagics, separately) who underwent pharyngeal or esophageal manometry studies with outcomes compared to young healthy controls (in healthy volunteer studies) or younger patients (in dysphagia).

### 2.1. Eligibility Criteria

Inclusion and exclusion criteria for studies are included as Table 1.

### 2.2. Participants

Definitions of aging vary. The definition used when referring to the older population is individuals aged 60 years of age and older. This definition is in keeping with the World Health Organization formal definition of older age [28], however an age of 65 and older is mostly in keeping with a majority view of the terms ‘aged’, ‘older’, ‘elderly’, or ‘geriatric’. Our original intention was to use 65 as a cut-off, however many important studies of age-related manometry changes used sixty as age cut-off and for this reason we concluded to use 60 years of age. The comparator was human participants between 18 and 59 years of age. 

### 2.3. Interventions

Participants had to undergo manometry using standard manometry equipment. Reports had to include details on the equipment used, technical details on sensor technology, sensor spacing, and catheter configuration and, in addition, participant posture, volume, consistency, and type of the boluses swallowed. 

### 2.4. Comparators

Normative values had to be either standardized for the equipment configuration or reported based upon inclusion of a young participant comparator group. 

### 2.5. Outcomes

There are no universally agreed criteria for the interpretation of pharyngeal manometry. For an interpretation of pharyngeal manometry related to functional outcomes such as pulmonary aspiration risk and pharyngeal residue see Cock & Omari [29]. 

The UES is tonically contracted, and needs to neurogenically deactivate to relax and open. UES resting or basal pressures give some indication of this basal tone. Another important aspect measured during pharyngeal manometry relates to opening of the UES, or cricopharyngeal/UES dysfunction [30,31] whereby UES opening is inadequate for the size/volume of the bolus swallowed due a non-opening and/or nonrelaxing UES high pressure zone. UES dysfunctions commonly result from neurogenic or myogenic causes affecting UES relaxation and UES opening extent [32,33,34]. Restricted opening commonly leads to increased intrabolus pressure above and pressure gradient across the sphincter, provided pharyngeal contractility is sufficiently propulsive [35]. Pharyngeal contractility is commonly reported as a peak pressure (PeakP) per sensor or average across a region. Some studies also reported the duration of the pharyngeal swallow. Combining both pressure and duration with length, pressure “contractile integrals” are also described per region, with a global “Pharyngeal contractile integral” (PhCI) [36].

In summary, the outcomes reported for pharyngeal studies were

1. Upper esophageal sphincter basal pressure (UES-BP in mmHg).
2. Upper esophageal sphincter relaxation
  - Duration (UES-RT)
  - Integrated relaxation pressure in 0.25 s (UES-IRP in mmHg)
3. UES opening extent on radiology or impedance base (UES Max Adm in milliSievert—mS)
4. Intrabolus pressure above sphincter (IBP in mmHg at 1 cm above UES).
5. Pharyngeal contractility—(PeakP or PhCI) and duration (milliseconds—ms)

As a broad principle the “classical criteria” were considered for conventional esophageal manometry studies [37] and the “Chicago classification criteria” for HRM [17,18,19]. As these criteria have gone through several iterations it was deemed reasonable if studies were reported by the prevalent criterion version at the time of the study. Esophagogastric junction (EGJ) barrier function including lower esophageal sphincter (LES) resting pressure and relaxation forms a critical component of the manometric assessment of esophageal function. Following on from this, distal esophageal contractility leads to the completion of bolus flow through the EGJ. Few studies specifically report on proximal esophageal contractility in older subjects [38,39]—no comprehensive assessment of this aspect was possible and more studies are needed. A few studies reported on esophageal peristaltic success.

In summary, outcomes reported for esophageal studies were as follows.

1. Esophagogastric junction barrier function (LES resting pressure in mmHg, EGJ contractile integral in mmHg.cm).
2. Lower esophageal sphincter relaxation pressure (integrated relaxation pressure in 4 s IRP4 in mmHg).
3. Contractility of the proximal esophagus (limited data) (proximal contractile integral/PCI—pressure × length × duration in mmHg.cm.s).
4. Contractility of the distal esophagus (as mean peak pressure in mmHg or distal contractile integral—pressure × length × duration in mmHg.cm.s).
5. Esophageal peristaltic success (% successful peristalsis).

### 2.6. Settings

There were no restrictions on the setting.

### 2.7. Language

English language articles were included. Articles in other languages were only included if a full translation in English was simultaneously published. 

### 2.8. Search Strategy & Data Management

A search was undertaken for English language articles dated 1948 to 2018 using the search terms manometry AND age/aging/elderly/older AND either pharynx/pharyngeal plus high-resolution or esophagus/esophageal. Studies of anorectal manometry were excluded. 

Cross-referencing and the author’s own collections were used to supplement the search strategy. 

#### 2.8.1. Information Sources

The literature search strategy was developed using medical subjects headings (MeSH) terms related to manometry in older subjects. Medline (OVID interface, 1948 onwards), Pubmed at https://www.ncbi.nlm.nih.gov/pubmed, and Web of Science core collection v5.29.

#### 2.8.2. Data Management and Selection Process

Records of all searches (titles only) were saved in a folder on a password protected and fire walled personal computer. Eligible (articles) were saved in PDF format in a shared folder and, where needed, printed out for reading. Titles, abstracts and, where necessary, article text was scanned to assess eligibility for inclusion if the study contained data on a participant group as defined (see Table 1). Searches were undertaken by author CC and screened for inclusion by authors CC and TO independently.

#### 2.8.3 Data Collection Process

Data reporting was specific for methodology during manometry. Differences in equipment (e.g., catheter specifications/diameter [39,40]) may account for different values for the same variable. Interpretation of data should be undertaken with this knowledge and as such, rather than performing a meta-analysis, “functional” interpretation was applied to the data (Table 2). 

#### 2.8.4. Data, Outcomes and Prioritization

Consideration was given to the functional and clinical relevance of findings. Pharyngeal and esophageal studies were grouped into those in healthy volunteers or symptomatic patients. Technical data on the analyses are included in Table 4.

#### 2.8.5. Risk of Bias

Bias was assessed as per Table 8.5 in the Cochrane Handbook for Systematic Reviews of Interventions at http://handbook-5-1.cochrane.org/. Possible bias was assessed for each of the six domains described: selection, performance, detection, attrition, reporting, and other sources of bias. Results for biases are included in the results section below.

#### 2.8.6. Data Synthesis

Due to heterogeneity in measurement techniques and the potential for catheter configuration or measurement technique to influence results, methodology was focused on regional changes related to functional swallowing outcomes.

Studies in patients (but not healthy volunteers) were rated for quality (very high to low from A–D) and strength of recommendation (strong or weak for or against) with the overriding question on whether the study results/outcomes were likely to change clinical management. An adaptation of the Grading of Recommendations Assessment, Development, and Evaluation (GRADE) scale for diagnostic tests, specifically adapted for esophageal manometry was applied (Table 2) [41,42]. Study quality was modified as described within GRADE [41,42]

## 3. Results

### 3.1. Literature Search and Study Characteristics

**Figure 1:**
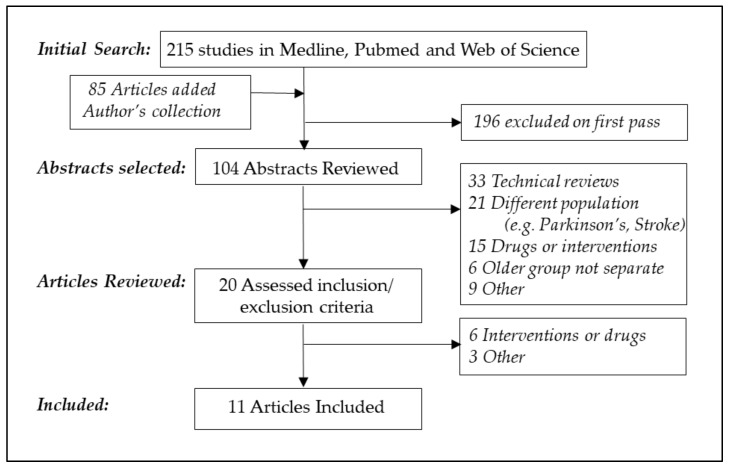
Search strategy for pharyngeal manometry in older persons.

**Figure 2:**
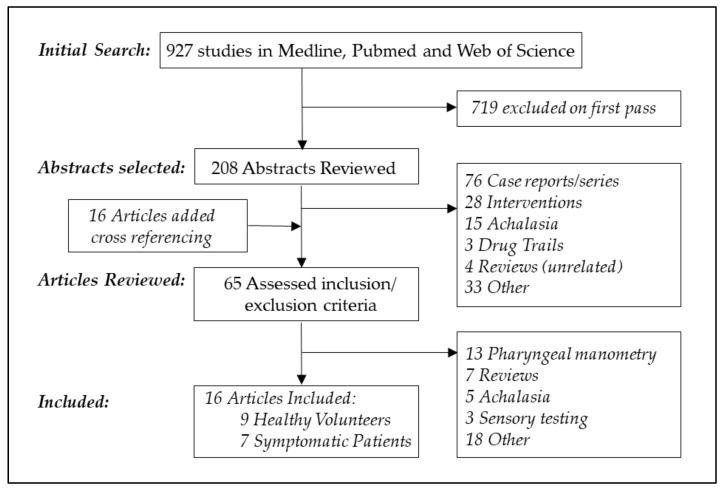
Search strategy for esophageal manometry in older persons.

The results of the literature search for pharyngeal manometry (Figure 1) and esophageal manometry (Figure 2) are reported in Figure 1 and Figure 2. Two hundred and fifteen studies of pharyngeal manometry and nine hundred and twenty seven studies of esophageal manometry were retrieved. During the “Web of Science” search, alternate possibilities such as “anorectal” were specifically excluded. Terms such as “aging” or “older” produced more focused results, as compared to broad search terms such as “age”.

### 3.2. Results of Manometry Studies

Eleven pharyngeal [10,34,39,43,44,45,46,47,48,49,50] and sixteen esophageal studies [23,24,25,51,52,53,54,55,56,57,58,59,60,61,62] were identified and results described in Table 3 (summary) and Table 4 (measurements).

### 3.3. Study Quality and Bias

Quality of six diagnostic studies (one pharyngeal, four esophageal, and one in both) between older and young cohorts are summarized in Table 5. No study achieved more than a moderate quality or strength of recommendation for diagnostic manometry in older people. 

## 4. Discussion

Based on this systematic review, the dominant age-related changes in swallowing physiology include (i) greater UES restriction, (ii) increased pharyngeal contractility, (iii) decreased distal esophageal contractility, and (iv) reduced LES relaxation. Major esophageal motility disorders, achalasia, and distal esophageal spasm in particular, may be more prevalent with age.

Abnormalities of UES relaxation and opening have been repeatedly reported in both healthy volunteers and dysphagia patients of advanced age. Associated features include increased hypopharyngeal intrabolus pressure, a biomechanical consequence of restriction, and increased pharyngeal contractility which may be compensatory response to restriction. Some authors have postulated decreased sphincter compliance [32,46]; and there is limited evidence suggesting reduced UES relaxation [34,39,47,48]. In contrast, dysphagia patient data suggests decompensation of swallowing indicated by weaker pharyngeal contractility with age. Readers are referred to a review of pharyngeal manometry by Cock and Omari [29].

Data on LES resting pressure are inconsistent, with different studies showing lower, higher, and unaltered LES pressure. However data on reduced LES relaxation with age are more reliable, particularly for subjects over 80 years. Data on esophageal contractility suggests reduced peristaltic amplitude with age contributes to a greater likelihood of peristaltic failure. When major motility disorders have been reported, achalasia and spastic esophageal motility were the most common diagnoses. Age-related loss of central and/or enteric nervous system functions are a likely cause of these changes [63]. Changes in esophageal compliance have been shown with aging, which may relate to loss of elastic tissues, or neuromuscular changes [64]. Such changes may contribute to the esophageal changes seen in our review. Readers are also referred to more recent reviews by Gyawali et al. [22] and Carlson and Pandolfino [65] on HRM and esophageal pressure topography in clinical practice. 

Our review identified very few clinical studies reporting manometry findings in older dysphagia patients. Given the burgeoning aging population in developing countries, more studies of older patient groups are needed to address this knowledge gap. Future studies should also focus attention on patients and subjects that are older than 85 years of age (the so-called “older old”) as data available suggests this as the threshold for manifestations of the most extreme forms of pharyngo-esophageal dysfunction. 

### Limitations

Whilst our search strategy identified many papers, some relevant studies may have been missed because inclusion of older patients was not mentioned in the title or listed in keywords. We did assess several papers which clearly contained data gathered in older subjects, but in which results for the older portion of the cohort were not distinguishable. Some studies were also excluded because they included subjects aged below our applied threshold of sixty years. Some studies tended to focus on certain aspects, such as lower esophageal sphincter relaxation, whilst omitting description of other features. Supplementary data tables may be one way for authors to address the need for clarity and still provide a more comprehensive summary of their data. 

## 5. Conclusions

The aging process alone leads to changes in swallowing function, most notably UES restriction and esophageal dysmotility. More clinical studies, across the older age range, and reporting consistent biomechanical endpoints, are needed. 

## Figures and Tables

**Table 1:** Inclusion and exclusion criteria.

Inclusion Criteria	Exclusion Criteria
Case control, Cohort, and Observational.	RCT (drug trails and therapeutic interventions), Review, Cases, and Case series.
At least one group ≥ 60 years of age.	Study focused on single disease process e.g., achalasia
Either healthy volunteers or a patient population with dysphagia included.	Surgery or radiotherapy involving the pharynx, UES or esophagus
Technical details of manometry procedure described.	Anorectal manometry
For pharyngeal studies the use of solid state sensors, 3 cm or less (“low-resolution”) or spaced at 1 cm or less “high-resolution” (HRPM).	For pharyngeal studies sensor spacing less than 3 cm
For esophageal studies both “low-resolution” (> 1 cm sensor spacing) and “high-resolution” (<1 cm or less) without or with impedance (HRM/HRIM).	Language other than English (LOTE) without available translation (simultaneous publication of English translation for LOTE articles).

**Table 2:** Grading of Recommendations Assessment Development and Evaluation (GRADE) applied to manometry studies

Quality of Evidence	Strength of Recommendation
High quality (A) e.g., High Resolution Manometry	Strong recommendation for (1)/↑↑
Moderate Quality (B)	Weak recommendation for (2)/↑
Low Quality (C) e.g., Low Resolution Manometry	Weak recommendation against (2)/↓
Very low quality (D)	Strong recommendation against (1)/↓↓

**Table 3:** Studies of pharyngeal and esophageal manometry in older persons.

Study	Population	Methods	Main Findings in Older
Pharyngeal Manometry			
Shaker R et al. Effect of aging and bolus variables on pharyngeal and upper esophageal sphincter motor function. Am J Physiol 1993; 264:G427–G432 [43].	Older (aged 76 ± 1.5 years) n = 12 Younger (aged 25 ± 1 years) n = 14 Healthy Volunteers	Videomanometry Gaeltec MMI spaced 1.5 cm	UES resting pressure lower Hypopharyngeal peak pressures increased Duration of hypopharyngeal pressure increased
Dejaeger E et al. Manofluorographic Analysis of Swallwing in the Elderly. Dysphagia 1994; 9:156–161 [44].	Older (aged 80 ± 5 years) n = 16 Younger (aged 28 ± 8 years) n = 20 Healthy Volunteers	Video manometry Tranducers at 4 cm, 1.5 cm intervals	Incomplete UES relaxation in 18% Less negative pressure at UES in older
McKee GJ et al. Does age and sex affect pharyngeal swallowing? Clin Otolaryngol 1998; 23:100–106 [45].	Older (60–85 years) n = 37 Younger (21–40 years) n = 36 Healthy Volunteers	Manometry 2 cm spacing Konigsberg	UES resting pressure lower UES opening earlier when referenced to UES closure (i.e., longer duration of UES relaxation) Less generation of negative pressure at the UES in older
Kern M et al. Comparison of Upper Esophegeal Sphincter Opening in Healthy Asymptomatic Young and Elderly Volunteers. Ann Otol Rhinol Laryngol 1999; 108:982–989 [46].	Older (75 ± 2.8 years) n = 14 Younger (32 ± 2.7 years) n = 14 Healthy Volunteers	Videomanometry Gaeltec MMI spaced 1.5 cm 5 & 10 mL liquid barium boluses	Duration of UES opening longer Duration UES maximally relaxed longer Significantly higher IBP above UES (5 & 10 mL)UES opening decreased (in AP diameter for 5 mL)
*Meier-Ewert HK et al. Effect of Age on Differences in Upper Esophageal Sphincter and Pharynx Pressures Between Patients With Dysphagia and Control Subjects. Am J Gastroenterol 2002; 96:35–40 [47].	Healthy Volunteers:Older (61–91 years) n = 15 Younger (32–59 years) n = 18 Patients:Older (60–88 years) n= 26 Younger (32–58 years) n = 15	Manometry Konigsberg 1.5 cm/2 cm	UES resting pressure lower (significant in controls) Increased UES residual pressure during solid bolus swallows only in healthy volunteers Decreased pharyngeal peak pressure during solid bolus swallows only in patients
Van Herwaarden MA, et al. Are Manometric Parameters of the Upper Esophageal Sphincter and Pharynx Affected by Age and Gender? Dysphagia 2003; 18:211–217 [48].	Older (>60 years) n = 23 Younger (<60 years) n = 61 Healthy Volunteers	Manometry Konigsberg 1.5 cm/2 cm	Decreasing UES resting pressure correlated with age (r = −0.41; *p* < 0.001) and lower UES residual pressure higher (liquids & solids) UES-RT shorter (liquids and solids); UES relaxation rate lower for all consistencies Pharyngeal amplitude increased Duration of contraction longer
Bardan E et al. Effect of aging on bolus kinematics during the pharyngeal phase of swallowing. Am J Physiol 2006; 290: G458-G465 [49].	Older (70–85 years) n = 8 Younger (18–40 years) n = 8	Videomanometry Study focused on bolus kinematics.	Bolus head (but not the bolus tail) slows significantly in the region between the epiglottis and UES only in older Negative pressure at the UES occurred less often: 41 vs. 53% for liquids (n.s.) and 55 vs. 83% of solids (*p* = 0.02)
Nativ-Zetzer et al. Pressure topography metrics for high-resolution pharyngeal-esophageal manofluorography—a normative study of younger and older adults. Neurogastroenterol Motil 2016; 28(5):721–731 [39].	Older (aged 60–80 years) n = 22 Younger (aged 21–40 years) n = 22	High-resolution manometry Manoscan 4.2 & 2.75 mm diameter catheters	Contractile integrals: PhCI, VPCI, TBI, and HPCI significantly greater (*p* < 0.05) Integrated UES relaxation pressure (UES-IRP) greater (*p* < 0.05) for all bolus trials. Proximal esophageal contraction (PCI) reduced
Cock et al. Maximum upper esophageal sphincter (UES) admittance: a non-specific marker of UES dysfunction. Neurogastroenterol Motil. 2016; 28:225–233 [34].	Older (≥80 years) n=16 Younger (<60 years) n = 50 Also CPB (n = 11) & MND (n = 16) groups included	High-resolution manometry MMS Unisensor	UES admittance (opening extent) reduced UES IRP higher with age (liquid only) Duration of pharyngeal bolus presence during and following swallow (residue) increased Swallow risk index (aspiration risk) increased
Yoon et al. Videofluoroscopic and Manometric Evaluationof Pharyngeal and Upper Esophageal Sphincter Function During Swallowing J Neurogastroenterol Motil, Vol. 20 No. 3 July, 2014 [50].	26 asymptomatic volunteers (12 men and 14 women; age, 19–81 years). Correlation with age reported.	High-resolution manometry Given Imaging	A significant correlation was shown between decreasing hypopharyngeal CI vs. age Decreasing median intrabolus pressure at UES vs. age Decreasing nadir pressure at UES vs. Age
Omari et al. Swallowing dysfunction in healthy older people using pharyngeal pressure-flow analysis. Neurogastroenterol Motil 2014, 26:59–68 [10].	Two older groups included 60–79 years (n = 18) & 80 + y (n = 20)	High-resolution manometry MMS Unisensor	Documented decrease in swallow function with pressure-flow parameters Increased SRI and increased PSIR Increased Flow Interval (Bolus Presence Time), Increased Nadir Impedance Correlations also of age vs. IBP (liquid)
Esophageal Manometry			
Healthy Volunteers			
Cock et al. Age-related impairment of esophagogastric junction relaxation and bolus flow time. World J Gastroenterol 2017; 23(15):2785–2794 [51]	Older (≥80 years) n = 15 Young (<60 years) n = 30 Asymptomatic volunteersGERD excluded by questionnaire	High-resolution impedance manometry (HRIM) MMS + Unisensor 5 and 10 mL liquid and viscous boluses in upright posture	Lower esophageal sphincter (LES) relaxation impaired (IRP4 11. 9 ± 2.3 vs. 5.9 ± 1.0 mmHg; *p* = 0.02). Bolus flow time through LES reduced (1.7 ± 0.3 vs. 3.8 ± 0.2 s; *p* < 0.001). Gastric resting pressure higher (9.4 ± 1.6 vs. 2.2 ± 1.5 mmHg). A novel index of LES contractility EGJ-contractile integral (contractility over three respiratory cycles at rest) similar in older
Cock et al. Impaired bolus clearance in asymptomatic older adults during high-resolution impedance manometry, Neurogastroenterol Motil 2016; 28(12):1890-1901 [52].	Older (≥80 years) n = 15 Young (<60 years) n = 30 Asymptomatic volunteersGERD excluded by questionnaire	High-resolution impedance manometry (HRIM) MMS + Unisensor 5 and 10 mL liquid and viscous boluses in sitting posture	Overall average Chicago classification metrics were similar Higher proportion unsuccessful bolus transit for both liquids (60 vs. 80%) and viscous (40 vs. 80%) Failed bolus transit associated with reduced contractility and longer peristaltic breaks
Besanko et al. Changes in Esophageal and Lower Esophageal Sphincter Motility with Healthy Aging [53].	Older ( ≥65 years) n = 10 Younger (<40 years) n = 10	Low-resolution Water perfused Dentsleeve; Trace!	Reduced lower esophageal relaxation in older group in supine, as well as upright posture and with increased bolus consistencies. Trend towards lower LES resting pressure
Dantas et al. Effect of Age on Proximal Esophageal Response to Swallowing. Arq Gastroenterol 2010 Oct-Dec; 47(4)339–343 [38].	Group I (18–30 years) n = 20 Group II (31–50 years) n = 27 Group III (51–74years) n = 22 Group C (III aged 51–59 years) n = 14 Group D (III aged ≥ 60 years) n = 8	Low-resolution Medizintechnik Polygram Upper GI	No difference in amplitude. Duration longer in youngest group Trend towards lower amplitude in group aged over 60 years of age (not statistically significant)
Grande et al. Deterioration of Esophageal Motility With Age: A Manometric Study of 79 Healthy Subjects. Am J Gastroenterol 1999; 94(7): 1795–1801 [54].	Six age cohorts (total n = 79) Sixth age cohort aged ≥ 65 years n = 13	Low-resolution Arndorfer, Beckman instruments	LES resting pressure reduced LES overall length increased UES pressure and length reduced Maximum peristaltic wave amplitude reduced in the distal (but not significantly proximal) esophagus Simultaneous contractions occurred more commonly in older subjects
Ferriolli et al. Aging, Esophageal Motility, and Gastroesophageal Reflux. J Am Geriatric Soc 1998; 46:1534–1537 [55].	Group I (20–30 years) n = 20 Group II (50–60 years) n = 10 Group III (70–80 years) n = 10 Healthy volunteers	Low-resolution Narco Bio 5 mL liquid and viscous boluses supine	LES resting pressure similar Contractile metrics similar Increased frequency of impaired peristalsis Clearance of scintigraphic reflux decreased
Nishimura et al. Effect of Aging on the Esophageal Motor Functions. J Smooth Muscle Res 1996; 32:43–50 [56].	Group 1 (<50 years) n = 11 Group 2 (50–59 years) n = 15 Group 3 (60–69 years) n = 11 Group 4 (≥70 years) n = 10	Low-resolution Arndorfer 3–5 mL liquids, supine	Trend towards lower LES resting pressure No difference in nadir LES pressure (relaxation) Lower proportion successful peristalsis ≥ 70 years Contractile amplitude reduced in ≥ 70 years
Richter et al. Esophageal Manometry in 95 Healthy Adult Volunteers. Dig Dis Sci 1987; 32:583–592 [57].	95 Healthy volunteers Older group (≥ 60 years) n = 13	Low-resolution Arndorfer Beckman instruments 5 mL liquids, supine	No difference in LES resting pressure Contractile amplitudes similar Duration contraction longer
Khan et al. Esophageal Motility in the Elderly. Dig Dis 1977; 22(12):1049–1054 [58].	Older group (≥60 years) n = 49 Young group (<40 years) n = 43 Asymptomatic per questionnaires	Low-resolution Water perfused 5 mL liquids	No difference in LES resting pressure LES relaxation reduced (82.2% vs. 94.1%; *p* < 0.003) Reduced amplitude distal and upper esophagus Increased “disordered” contractions (25.3 vs. 8.2%; *p* < 0.001)
Dysphagia Patients			
Nakato et al. Age-Related Differences in Clinical Characteristics and Esophageal Motility in Patients with Dysphagia. Dysphagia 2017; 32:374–382 [59].	Group A (≥ 65 years) n = 47 Group B (45–65 years) n = 42 Group C (<45 years) n = 27 Dysphagia symptoms	High-resolution impedance manometry (HRIM) Sandhill	Overall average Chicago classification metrics were similar Major motility disorders occurred in 28% of older and 39% of younger dysphagia cases. No difference in diagnoses between groups.
Shim et al. Effects of Age on Esophageal Motility: Use of High-resolution Esophageal Impedance Manometry. J Neurogastroenterol Motil 2017; 23:229–236 [60].	Group A (≥65 years) n = 62 Group B (40–65 years) n = 185 Group C (<40 years) n = 32 All symptoms	High-resolution impedance manometry (HRIM) Sandhill	Overall average Chicago classification metrics were similar Upper esophageal sphincter resting pressures measured and reported to be lower in older (Group A 63.8 mmHg ± 32.2 vs. Group B 92.5 ± 49 mmHg and Group C 92.7 ± 46.0 mmHg; *p* < 0.001)
Besanko et al. Lower esophageal sphincter relaxation is impaired in older patients with dysphagia World J Gastroenterol 2011; 17(10):1326–1331 [61].	Older group (≥80 years) n = 19 Young group (<50 years) n = 19 Dysphagia symptoms Achalasia excluded	Low-resolution Water perfused Dentsleeve; Trace! 5 mL liquids, solids Left lateral, upright	Resting LES pressure higher (23.4 ± 3.8 vs 14.9 ± 1.2 mmHg; *p* < 0.05) Nadir LES pressure higher 2.3 ± 0.6 vs. 0.7 ± 0.6 mmHg; *p* < 0.05) Restitution of LES earlier Amplitude and duration of contractions similar
Andrews et al. Age and gender affect likely manometric diagnosis: Audit of a tertiary referral hospital clinical esophageal manometry service. J Gastroenterol Hepatol 2009; 24:125–128 [24].	Older group (≥65 years) n = 135 Young (n = 317): Group 1 (17–24 years) n = 14 Group 2 (25–44 years) n = 87 Group 3 (45–59 years) n = 216 All symptoms	Low-resolution Water perfused Dentsleeve; Trace! 5 mL liquids, solidsLeft lateral, upright	Increased abnormal studies (79% vs. 57%; *p* = 0.013) Trend towards increased spastic type motility (*p* = 0.06)
Andrews et al. Is esophageal dysphagia in the extreme elderly (≥ 80 years) different to dysphagia in younger adults? A clinical manometry service audit. Dis Esophagus 2008; 21:656–659 [62].	Older group (≥80 years) n = 23 Young group (<50 years) n = 23 Dysphagia symptoms	Low-resolution Water perfused Dentsleeve; Trace! 5 mL liquids, solids Left lateral, upright	Resting LES pressure higher (26.1 ± 3.7 vs 16.8 ± 1.9 mmHg; *p* = 0.03) Increased failed peristalsis (63 vs. 32%; *p* = 0.006) Manometric diagnoses similar Fewer with heartburn symptom in addition
Robson & Glick. Dysphagia and Advancing Age. Are Manometric Abnormalities More Common in Older Patients? Dig Dis Sci 2003; 48(9): 1709–1712 [25].	Older group (≥65 years) n = 53 Young group (18–45 years) n = 53 Dysphagia symptoms	Low-resolutionWater perfused Medtronic5 mL liquids, supine	Equal number of abnormal studies (82% vs. 77%; *p* = NS) and achalasia diagnoses (32% vs. 34%; *p* = NS) LES resting pressure, relaxation and esophageal contractility similar. Peristaltic failure in 53% older and 40% young (*p* = NS)
Ribeiro et al. Esophageal Manometry: A Comparison of Findings in Younger and Older Patients. Am J Gastroenterol 1998; 93:706–710 [23].	Older Group (≥75 years) n = 66 Young (≤50 years) n = 122 All symptoms	Low-resolution Solid state Konigsberg 5 mL liquids	Dysphagia more common reason for referral LES resting pressure similar (28.6 mmHg vs. 27.2 mmHg). LES length similar. Peristaltic failure in 37% vs. 22% (*p* < 0.005) Amplitude of contractions similar More simultaneous contractions (15 vs. 4%; *p* < 0.02)Lower UES resting pressure (49.6 vs. 77.4 mmHg; *p* < 0.002) and less negative residual pressure Older patients more likely to have achalasia (15.2 vs. 4.1%; *p* < 0.05) or spastic disorders (16.6 vs. 5%; *p* < 0.05) Incomplete LES relaxation less

**Table 4:** Summary of measurement results for esophageal and pharyngeal manometry in older persons. Average values with SEM (ave ± sem) or median values with 25th and 75th percentiles: (med (25th; 75th)). NS = non-significant.

Study	Metric	Older	Younger	*p*-Value	Interpretation (Older Group Relative to Younger Group)
Upper Esophageal Sphincter Function					
UES Resting Pressure					
Shaker et al. 1993 [43]	UES-RP (mmHg)	43 ± 5	71 ± 8	<0.01	Lower UES resting pressure
Mc Kee et al. 1997 [45]	UES-RP (mmHg)	44	70	<0.001	Lower UES resting pressure
Meier-Ewert et al. 2001 (healthy volunteers) [47]	UES-RP (mmHg)	52 ± 6	86 ± 9	<0.05	Lower UES resting pressure
Van Herwaarden et al. 2003 [48]	UES-RP (mmHg)	46(20;116)	78(34;164)	<0.001	Lower UES resting pressure
Meier-Ewert et al. 2001 (patients) [47]	UES-RP (mmHg)	65 ± 9	96 ± 15	NS	Similar UES resting pressure
Intrabolus Pressure above UES (5 mL Liquids)					
Kern et al. 1999 [46]	Hypopharyngeal IBP	14 ± 2	7 ± 1	<0.05	Higher
Omari et al. 2014 [10]	Mean Pharyngeal IBP	10(4;30)	7(2;13)	NS	Similar
Cock et al. 2016 [34]	Mean Pharyngeal IBP	17(9;33)	10(5;16)	<0.05	Higher
UES Relaxation pressures (5 mL Liquids)					
Meier-Ewert et al. 2001 (healthy volunteers) [47]	UES residual pressure (mmHg)	5.1 ± 1.2	7.4 ± 2.7	NS	Similar residual pressure
Meier-Ewert et al. 2001 (patients) [47]	UES residual pressure (mmHg)	3.5 ± 1.5	−0.4 ± 3.5	NS	Similar residual pressure
Van Herwaarden et al. 2003 [48]	UES residual pressure (mmHg)	2.5(−8.4 to 14.5)	−3(−9.6 to 12)	<0.01	Higher residual pressure Decreased extent UES relaxation
Cock et al. 2016 [34]	UES-IRP (mmHg)	6(-1;23)	3(1;9)	NS	Similar extent relaxation
Nativ-Zeltzer et al. 2016 [39]	UES-IRP (mmHg)	4 ± 6	-3 ± 4	<0.05	Decreased extent UES relaxation
Duration of UES relaxation/opening (5 mL Liquids)					
Kern et al. 1999 [46]	Total duration UES opening Maximum opening	612 ± 9 ms 166 ± 14 ms (27%)	571 ± 8 ms 128 ± 12 ms (22%)	<0.05 <0.05	Increased duration UES relaxation
Meier-Ewert et al. 2001 (healthy volunteers) [47]	UES-RT (ms)	554 ± 47	605 ± 38	NS	Similar relaxation time
Meier-Ewert et al. 2001 (patients) [47]	UES-RT (ms)	525 ± 35	470 ± 39	<0.05	Increased duration UES relaxation
Van Herwaarden et al. 2003 [48]	UES relaxation time (50% drop and return to 50% baseline)	221 (75 to 379)	260 (133 to 535)	< 0.05	Decreased duration below 50% of baseline
UES Opening Extent (5 mL Liquids)					
Kern et al. 1999 [46]	Lateral projection/AP diameter (mm) AP projection/Lateral diameter (mm)	11 ± 0.4 21 ± 4	12.6 ± 0.6 20 ± 5	<0.05 NS	Decreased AP opening extent
Cock et al. 2016 [34]	UES Max Adm (mS)	3.8(2.9;4.2)	4.1(3.8;4.3)	<0.05	Decreased opening extent
UES postswallow Contractility (5 mL Liquids)					
Nativ-Zeltzer et al. 2016 [39]	UES-CI (mmHg.cm.s) UES-PeakP (mmHg)	405 ± 170 214 ± 72	408 ± 170 205 ± 46	NS NS	Similar postswallow UES contractility
Pharyngeal Contractility (5 mL Liquids)					
Shaker et al. 1993 [43]	Hypopharyngeal PeakP (mmHg) Duration hypopharynx (ms)	196 ± 12 437 ± 69	137 ± 9 204 ± 21	<0.01 <0.01	Increased hypopharyngeal contractile vigor and duration
Meier-Ewert et al. 2001 (healthy volunteers) [47].	Pharyngeal PeakP (mmHg) Duration pharyngeal contraction (ms)	182 ± 20 763 ± 64	139 ± 13 593 ± 55	NS NS	Similar pharyngeal contractility
Meier-Ewert et al. 2001 (patients) [47].	Pharyngeal PeakP (mmHg) Duration pharyngeal contraction (ms)	96 ± 15 712 ± 64	144 ± 21 712 ± 58	<0.05 NS	Decreased contractile vigor in patients
Van Herwaarden et al. 2003 [48].	Pharyngeal PeakP (mmHg) Duration pharyngeal contraction (ms)	152(44 to 379) 448(324 to 835)	133(53 to 220) 396(187 to 628)	<0.05 <0.05	Increased pharyngeal contractile vigor and duration
Omari et al 2014 [10].	Mean Pharyngeal PeakP (mmHg)	145(108;194)	132(103;213)	NS	Similar pharyngeal contractility
Cock et al. 2016 [34].	Mean Pharyngeal PeakP (mmHg)	161(117;221)	136(104;208)	NS	Similar pharyngeal contractility
Nativ-Zeltzer et al. 2016 [39]	P-max (PeakP) (mmHg) PhCI (mmHg.cm.s)	249 ± 54 363 ± 110	211 ± 64 256 ± 84	<0.05 <0.05	Increased pharyngeal contractility
Esophageal Studies:					
Esophagogastric junction (EGJ) barrier function					
Healthy Volunteers					
Cock et al. 2017 [51]	EGJ contractile integral for 3 respiratory cycles (mmHg.cm)	34 ± 5	25 ± 5	NS	Similar EGJ-CI
Besanko et al. 2014 [53]	Lower esophageal sphincter resting pressure (LES-RP) (mmHg)	16 ± 3	21 ± 1	0.08	Lower (trend) LES-RP
Grande et al. 1999 [54]	LES-RP (mmHg)	11–25	16–38	<0.001	Lower LES-RP
Ferrioli et al. 1998 [55]	LES-RP (mmHg)	35 ± 9	31 ± 14	NS	Similar LES-RP
Nishimura et al. 1996 [56]	LES-RP (mmHg)	15(8;27)	11(4;16)	NS	Similar LES-RP
Dysphagia Patients					
Besanko et al. 2011 [61]	LES-RP (mmHg)	23 ± 4	15 ± 1	<0.05	Higher LES-RP
Andrews et al. 2008 [62]	LES-RP (mmHg)	26 ± 4	17 ± 2	0.03	Higher LES-RP
Robson et al. 2003 [25]	LES-RP (mmHg)	33.3	32.5	NS	Similar LES-RP
Lower esophageal sphincter (LES) relaxation					
Healthy Volunteers					
Cock et al. 2017 [51]	4-second Integrated Relaxation Pressure (IRP4) (mmHg)	12 ± 2 (Liquid) 14 ± 2 (Viscous)	6 ± 1 (L) 7 ± 1 (V)	0.02 0.02	Decreased LES relaxation(Upright)
Cock et al. 2016 [52]	IRP4 (mmHg)	9 ± 2 (L) 15 ± 2 (V)	8 ± 1 (L) 8 ± 1 (V)	NS 0.002	LES relaxation similar for liquids, but decreased for increased consistency (upright)
Besanko et al. 2014 [53]	IRP4 (mmHg)	4 ± 1 (Right Lateral) 7 ± 1 (Upright Liquid) 8 ± 1 (Upright Solids)	3 ± 1 (RL) 3 ± 1 (UL) 4 ± 1 (US)	NS <0.01 <0.001	Decreased LES relaxation(upright)
Dysphagia Patients					
Nakato et al. 2017 [59]	IRP4 (mmHg)	14 (8–27)	17 (9–30)	NS	Similar LES relaxation
Besanko et al. 2011 [61]	Nadir LES pressure (mmHg)	2.3 ± 0.6	0.7 ± 0.6	<0.05	Decreased LES relaxation
Robson et al. 2003 [25]	Proportion complete relaxation (%)	24/53 (45)	23/53 (43)	NS	Similar LES relaxation
Esophageal Contractility					
Healthy Volunteers					
Cock et al. 2016 [52]	Distal esophageal peak pressure (PeakP) (mmHg) Distal Contractile Integral (DCI) (mmHg.cm.s)	56 ± 9 729 ± 224	66 ± 9 766 ± 123	NS NS	Similar peak pressure and DCI
Besanko et al. 2014 [53]	Peak P (mmHg) DCI (mmHg.cm.s)	38 ± 9 835 ± 260	41 ± 8 947 ± 201	NS NS	Similar peak pressure and DCI
Grande et al. 1999 [54]	Distal amplitude (mmHg)	40–77	56–158	<0.001	Lower mean distal amplitude
Ferrioli et al. 1998 [55]	Contractile amplitude (mmHg)	97 ± 41	107 ± 35	NS	Similar mean distal amplitude
Nishimura et al. 1996 [56]	5 cm above LES (mmHg)	37 (20;54)	114 (58;142)	<0.05	Lower mean distal amplitude
Dysphagia Patients					
Nakato et al. 2017 [59]	DCI (mmHg.cm.s)	1005 (350;2063)	464 (218–1227)	NS	Similar DCI
Besanko et al. 2011 [61]	Peak P (mmHg)	54 ± 8	62 ± 6	NS	Similar peak pressure and DCI
Robson et al. 2003 [25]	Contractile amplitude (mmHg)	71	74	NS	Similar mean distal amplitude
Esophageal Peristalsis (Success)					
Healthy Volunteers					
Cock et al. 2016 [52]	Percent successful peristaltic contractions (%)	60 (Liquids) 40 (Viscous)	82 (L) 83 (V)	<0.05	Decrease in successful peristalsis
Nishimura et al. 1996 [56]	Percent successful peristaltic contractions (%)	80 (60;100) Liquids	100 (90;100) (L)	<0.05	Decrease in successful peristalsis
Dysphagia Patients—no data					

**Table 5:** Quality and strength of recommendations for diagnostic manometry studies.

Study	Comparative Diagnostic	GRADE Recommendation
***Pharyngeal Studies in Dysphagia Patients***		
Ribeiro et al. Esophageal Manometry: A Comparison of Findings in Younger and Older Patients. Am J Gastroenterol 1998; 93:706–710 [23].	Increase in “abnormal” studies	2B
Meier-Ewert HK et al. Effect of Age on Differences in Upper Esophageal Sphincter and Pharynx Pressures Between Patients With Dysphagia and Control Subjects. Am J Gastroenterol 2002; 96:35–40 [47].	Different mechanism	2B
**Esophageal Studies in Dysphagia Patients**		
Nakato et al. Age-Related Differences in Clinical Characteristics and Esophageal Motility in Patients with Dysphagia. Dysphagia 2017; 32:374–382 [59].	Major diagnosis in 39 vs. 28%	2B
Shim et al. Effects of Age on Esophageal Motility: Use of High-resolution Esophageal Impedance Manometry. J Neurogastroenterol Motil 2017; 23:229–236 [60].	Similar numbers	2C
Andrews et al. Age and gender affect likely manometric diagnosis: Audit of a tertiary referral hospital clinical esophageal manometry service. J Gastroenterol Hepatol 2009; 24:125–128 [24].	Increase in “abnormal” studies	2C
Robson & Glick. Dysphagia and Advancing Age. Are Manometric Abnormalities More Common in Older Patients? Dig Dis Sci 2003; 48(9): 1709–1712 [25].	High proportion achalasia	2B
Ribeiro et al. Esophageal Manometry: A Comparison of Findings in Younger and Older Patients. Am J Gastroenterol 1998; 93:706–710 [23].	Increase in “abnormal” studies	2B

The risk of bias in studies of esophageal or pharyngeal manometry in healthy volunteers/patients was considered low overall.
